# UHRF1/UBE2L6/UBR4-mediated ubiquitination regulates EZH2 abundance and thereby melanocytic differentiation phenotypes in melanoma

**DOI:** 10.1038/s41388-023-02631-8

**Published:** 2023-03-11

**Authors:** Gamze Kuser-Abali, Youfang Zhang, Pacman Szeto, Peinan Zhao, Samar Masoumi-Moghaddam, Clare G. Fedele, Isobel Leece, Cheng Huang, Jen G. Cheung, Malaka Ameratunga, Fumihito Noguchi, Miles C. Andrews, Nicholas C. Wong, Ralf B. Schittenhelm, Mark Shackleton

**Affiliations:** 1 Central Clinical School, Monash University, Melbourne, VIC Australia; 2 Alfred Health, Melbourne, VIC Australia; 3 CSL Limited, Melbourne, VIC Australia; 4 Monash Proteomics and Metabolomics Facility and the Department of Biochemistry and Molecular Biology, Biomedicine Discovery Institute, Monash University, Melbourne, VIC Australia; 5 Monash Bioinformatics Platform, Monash University, Melbourne, VIC Australia

**Keywords:** Melanoma, Ubiquitylation

## Abstract

Cellular heterogeneity in cancer is linked to disease progression and therapy response, although mechanisms regulating distinct cellular states within tumors are not well understood. We identified melanin pigment content as a major source of cellular heterogeneity in melanoma and compared RNAseq data from high-pigmented (HPCs) and low-pigmented melanoma cells (LPCs), suggesting EZH2 as a master regulator of these states. EZH2 protein was found to be upregulated in LPCs and inversely correlated with melanin deposition in pigmented patient melanomas. Surprisingly, conventional EZH2 methyltransferase inhibitors, GSK126 and EPZ6438, had no effect on LPC survival, clonogenicity and pigmentation, despite fully inhibiting methyltransferase activity. In contrast, EZH2 silencing by siRNA or degradation by DZNep or MS1943 inhibited growth of LPCs and induced HPCs. As the proteasomal inhibitor MG132 induced EZH2 protein in HPCs, we evaluated ubiquitin pathway proteins in HPC vs LPCs. Biochemical assays and animal studies demonstrated that in LPCs, the E2-conjugating enzyme UBE2L6 depletes EZH2 protein in cooperation with UBR4, an E3 ligase, via ubiquitination at EZH2’s K381 residue, and is downregulated in LPCs by UHRF1-mediated CpG methylation. Targeting UHRF1/UBE2L6/UBR4-mediated regulation of EZH2 offers potential for modulating the activity of this oncoprotein in contexts in which conventional EZH2 methyltransferase inhibitors are ineffective.

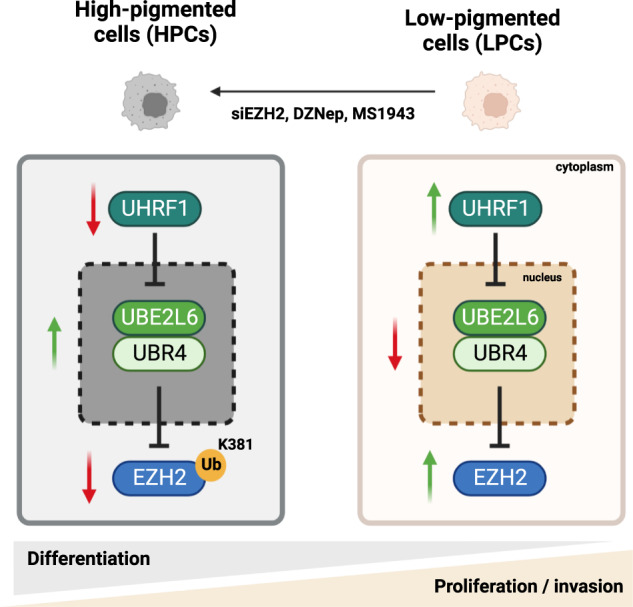

## Introduction

The ability of cancer cells to adapt, survive and proliferate during tumor growth, metastasis, and treatment is a major impediment to improving patient outcomes. As the same mechanisms that enable cancer cell adaptation also fuel intratumoral heterogeneity (ITH), understanding drivers of ITH is a high priority in cancer research [1].

ITH is facilitated by phenotype switching, in which individual cells reversibly switch via epigenetic mechanisms between different transcriptional programs, functional states and differentiation phenotypes [2–4]. In melanoma, switching between invasive and proliferative states was shown to be regulated by enhancer of zeste homolog 2 (EZH2) [3, 4], with the proliferative state characterized by high expression of the melanocyte transcription factor MITF and low expression of BRN2 and AXL, and opposite expression patterns found in the invasive state.

EZH2 suppresses gene transcription by catalyzing trimethylation of histone H3 at lysine 27 (H3K27me3), plays an important role in maintaining cells in a progenitor-like state through silencing genes associated with differentiation [5, 6], is aberrantly overexpressed in multiple cancers [5–12] and functions to promote tumor growth and metastasis in models of malignant disease [13]. EZH2 activation by mutation, gene amplification or increased transcription was noted in about 20% of cutaneous melanomas in the TCGA cohort (SKCM) [14, 15]. In clinical samples, EZH2 increased during disease evolution from benign nevus to metastatic melanoma [7, 16, 17]. In melanoma, EZH2 represses genes associated with tumor suppression, cell differentiation, cell cycle inhibition, metastasis, and antigen processing and presentation [7, 16, 17], and is upregulated transcriptionally by E2F and c-myc and suppressed by differentiation-promoting factors such as pRb and p16INK4b [18, 19]. MicroRNAs also downregulate expression of EZH2 in melanoma [20, 21]. Mechanisms of post-translational EZH2 regulation in melanoma are largely unknown.

Highly specific EZH2 methyltransferase inhibitors, GSK126 and EPZ-6438, are currently in clinical trials [13]. Although these have shown antitumor effects in lymphomas with enzyme-activating mutations of EZH2 [22, 23] and in ovarian cancer cells with inactivating mutations of ARID1A [24], some cancers appear resistant to EZH2 methyltransferase inhibition but sensitive to genetic depletion of EZH2, raising the possibility that EZH2 promotes tumorigenesis via methyltransferase-independent mechanisms. It has also been shown that EZH2 enzymatic inhibitors have better activity against EZH2 mutant melanoma cells than wild type cells [25]. Indeed, as EZH2 can promote cancer independently of its methyltransferase activity [25] or by acting as a transcriptional coactivator [26–28], targeting EZH2 abundance may be more effective than enzymatic EZH2 inhibition against cancers that are driven by EZH2 methyltransferase-independent mechanisms.

Cellular protein activity and stability are regulated by post-translational protein modifications [29] such as ubiquitination, which involve reversible addition of ubiquitin proteins to lysine residues of target substrates [30]. This is catalyzed by a series of enzymes: (1) ubiquitin-activating enzymes (E1) that use ATP to form ubiquitin-thioester bonds, (2) ubiquitin-conjugating enzymes (E2) that bind active ubiquitin to cysteine residues, and (3) ubiquitin-ligase enzymes (E3) that interact with E2 enzymes, catalyze formation of covalent bonds between ubiquitin and target substrates, and regulate substrate specificity. EZH2 protein undergoes ubiquitin-dependent degradation in different contexts by several E3 ligases, including β-TrCP, SMURF2, PRAJA1, MDM2 and FBW7, [31–35]. Recently, UBE2L6 was shown to be a tumor-suppressor and a prognostic marker in melanoma [36]. Here, we report UBE2L6 as an E2 ubiquitin-conjugating enzyme for EZH2 that regulates EZH2 abundance in melanoma cells.

## Materials and Methods

### Mice and tumors

Eight-week-old NOD SCID IL2R-/- mice (NSG) were injected subcutaneously with empty vector- or UBE2LB_pLX307-containing 28:B4:F3 melanoma cells (*n* = 11 mice per group). Tumors were measured weekly using calipers. Once a tumor reached 20 mm diameter, mice were sacrificed. All animal experiments were performed according to approved protocol E/1792/2018/M (AMREP Animal Ethics Committee of Monash University).

### Human melanoma tumor samples

19 pigmented and 20 non-pigmented human melanoma tissue sections were obtained from Melanoma Research Victoria (MRV).

### Cell lines, chemicals, plasmids, and siRNA

The cell lines, chemicals, plasmids, siRNAs, and qPCR primers used for treatment in this study are listed in Table S1.

For cell lines, mycoplasma tests were performed in our lab. Short tandem repeat (STR) profiling was done in the Australian Genome Research Facility (AGRF) to authenticate cell lines.

### Co-Immunoprecipitation, pulldown, and ubiquitination assays

Co-IP, pulldown and ubiquitination assays were done as described [32, 37].

### Western blot

Total proteins were extracted and analysed as described [37], using 4–20% Mini-PROTEAN TGX Stain-Free Protein Gels (BioRad, 4568096). PVDF membranes were incubated with respective antibodies outlined in Table S1. Signals were detected using Clarity ECL Western blotting Substrate (BioRad) and quantified by ImageJ.

### Quantitative PCR and Methylation-sensitive PCR (MSP)

Total RNA was extracted and underwent qPCR according to manufacturer’s instructions and as described [37]. RNA expression changes were determined using a ΔΔCt method [38]. Primers are listed in Table S1. Methylation of UBE2L6 promoters was analyzed as described [39].

### Cell cycle and apoptosis assays

Cells were fixed for cell cycle and apoptosis assays as described [37], and analysed by flow cytometry (FACSFusion).

### Cell Proliferation, clonogenicity and invasion assays

Cell proliferation, clonogenicity and invasion assays were performed as described [37].

### Cell senescence β-Gal assay

Cellular senescence was analyzed using, Senescence β-Galactosidase staining kits (Cell Signaling #9860).

### Extracellular melanin assay

For quantitative analysis of melanin, a modification of the method described by Huberman et al. was used [40].

### Immunofluorescence (IF)

Cultured or FACSorted cells were treated and stained with antibodies (Table S1) as described [32] for IF studies using Leica DM IL LED inverted fluorescent microscopy.

### Pigmentation-based flow cytometry sorting of PDX samples

Harvested tumours were dissociated into single cells and labelled as described [41]. Pigmentation was assessed as described [42].

### Immunohistochemistry (IHC), Fontana Masson and Schmorl’s staining

Spontaneous metastases in mice were evaluated by human mitochondrial IHC of liver, lung and lymph nodes [41]. IHC [37] and Fontana Masson and Schmorl’s staining [42] were performed as described. Antibodies used for IHC staining of EZH2 and UBE2L6 are listed in Table S1. Aperio ImageScope Software was used to evaluate IHC images.

### RNA-Seq data and gene set enrichment analysis

FASTQ files were processed via Laxy and the RNAsik pipeline. The GRCh38 reference genome was used for STAR alignment [43] and gene expression counts were performed using featureCounts [44]. Gene counts were analysed using Degust (https://zenodo.org/record/3501067) for differential expression (DE) analysis (limma voom). R package ClusterProfiler (v.4.0.0) was used for Gene Set Enrichment Analysis (GSEA) and over-representation analysis (ORA) [45]. For GESA, DE gene lists were ranked by logFC and the testing method was set to “fgsea”. For both GSEA and ORA, the following annotated gene sets were tested for significant enrichment: Gene Ontology (GO), Reactome, Hallmark and Oncogenic signatures. Genesets were downloaded from the Molecular signatures database (MSigDB) [46]. Genetic variants were identified using the GATK RNAseq variant discovery pipeline.

### The Cancer Genome Atlas (TCGA) survival analysis

Clinical data and mRNA expression profiles for skin melanoma samples in TCGA PanCancer Atlas database were accessed through the public cBioPortal (http://www.cbioportal.org) [47]. Using the R package ‘survminer’ 0.4.4 and ‘survival’ 3.1-11, respectively, overall survival (OS) Kaplan-Meier curves were plotted, and the log-rank test was performed.

### Liquid chromatography–tandem mass spectrometry (LC/MS) interactome and post-translational modification (PTM) analysis

For LC/MS interactome studies, a Dionex UltiMate 3000 RSLCnano system equipped with a Dionex UltiMate 3000 RS autosampler, an Acclaim PepMap RSLC analytical column and an Acclaim PepMap 100 trap column (Thermo Scientific) were used.

For label-free quantification (LFQ) analysis and protein identification, raw data files were analyzed using MaxQuant software suite v1.6.5.0 [48] against Andromeda search engine [49] and in-house standard parameters. Results were analyzed and visualized using LFQ-Analyst [50].

For PTM analysis, raw files were searched with Byonic v3.0.0 (ProteinMetrics) using GlyGly at lysine as a variable modification. Only peptides and proteins falling within a false discovery rate (FDR) of 1% based on a decoy database were further analyzed.

### Statistical analysis

Analyses were performed using GraphPad Prism version 7. Analyses were performed using log-rank tests, unpaired two-tailed t-tests, one-way ANOVA, or Tukey’s multiple comparison tests as appropriate to the data type. P values less than 0.05 were considered significant.

## Results

### Low pigmented melanoma cells demonstrate upregulation of EZH2 protein and EZH2-target genes

**Fig. 1:**
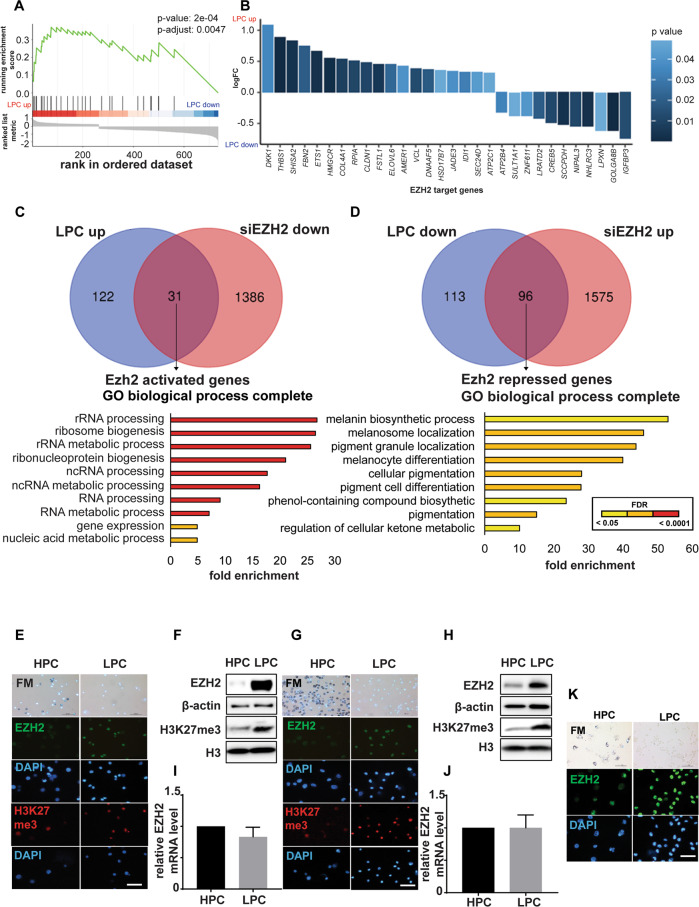
EZH2 protein, but not *EZH2* mRNA, is upregulated in LPCs. **A** Gene set enrichment analysis (GSEA) showing upregulated EZH2 targets in LPCs isolated from 28:B4:F3 cells. The EZH2 target genes were defined as down-regulated upon knockdown of EZH2 [51]. A positive normalized enrichment score (NES) indicates gene set enrichment at the top of the ranked list, which includes genes upregulated in LPC compared to HPC. **B**. EZH2 target genes that were differentially expressed between LPC and HCP. Positive logFC indicates upregulation in LPC. **C**, **D** Venn diagrams showing overlap of significantly upregulated (**C**) or downregulated (**D**) genes in B16-F10 LPCs relative to HPCs, and of siEzh2-downregulated (**C**) or upregulated (**D**) genes in parental B16-F10 cells relative to scramble controls (FDR-adjusted *p*-value < 0.05, upper panels). Lower bars show GO term enrichment analysis of the 31 (**C**) and 96 (**D**) common genes, showing enriched functions (FDR-adjusted *p*-value < 0.05, lower panel; colour indicates p-values adjusted by false discovery rate (FDR) via the Benjamini-Hochberg procedure). **E**–**J** Bright-field (BF) microscopy of Fontana-Masson staining (upper panel) and immunofluorescence (IF) images probed for EZH2 (green) and H3K27me3 (red) in HPCs and LPCs from 28:B4:F3 (**E**) and B16-F10 (**G**) cells. Nuclei shown by DAPI (blue; lower panels). Scale bars: 50 µm. To right of each set of images are shown endogenous EZH2 and H3K27me3 protein levels in HPCs and LPCs measured by western blot in (**F**) 28:B4:F3 and (**H**) B16-F10 cells, and *EZH2* mRNA levels in HPCs and LPCs in (**I**) 28:B4:F3 and (**J**) B16-F10 cells. **K** BF microscopy of Fontana-Masson staining (upper panel) and IF images probed for EZH2 (green) and H3K27me3 (red) in HPCs and LPCs from a pigmented patient-derived xenograft melanoma. Nuclei shown by DAPI (blue; lower panels). Scale bar: 50 µm. *n* = 3 biological replicates.

To interrogate functional differences amongst melanoma cells according to their levels of melanin pigment, RNAseq was performed on low pigmented cells (LPCs) and high pigmented cells (HPCs) purified from 28:F3:B4 cells by flow-cytometry [42]. GSEA analysis revealed that the LU-EZH2-target-DN gene set [51] was markedly enriched in LPCs (*p* = 2 × 10^−4^) (Fig. 1A, B). We thus also compared by GSEA differentially expressed gene signatures (DEGs) in sorted LPCs and HPCs from B16-F10 cells with those of EZH2 silenced B16-F10 cells (Table S2 and S3). Thirty-one of 153 genes upregulated in LPC were EZH2-activated genes (Fig. 1C, top panel Venn diagram) and 96 of 209 genes downregulated in LPCs were EZH2-repressed genes (Fig. 1D, top panel Venn diagram). While over-representation analysis (ORA) by Gene Ontology biological processes of the 31 EZH2-activated genes showed enrichment for ribosomal biogenesis (Fig. 1C, bottom panel and Table S4), the 96 EZH2-repressed genes were enriched in melanin biosynthesis pathways (Fig. 1D, bottom panel and Tables S5 and S6), including *Oca2*, which was one of the genes most negatively correlated (*p* = 0.002, Table S7) with *EZH2* in the TCGA SKCM. These data suggested that increased EZH2 activity in LPCs compared to HPCs might upregulate ribosomal biogenesis and/or suppress melanocytic differentiation and melanin synthesis.

To investigate this, we evaluated *EZH2* mRNA and EZH2 protein levels, as well as a marker of EZH2’s methyltransferase activity, H3K27me3, in HPCs and LPCs from 28:F3:B4, B16-F10 and C006-M1 pigmented melanoma cells (Fig. S1A–C). Western blotting and immunofluorescence (IF) staining demonstrated that EZH2 and H3K27me3 were higher in LPCs than HPCs (Fig. 1E–H and Fig. S1D). However, no significant difference was observed between LPCs and HPCs in *EZH2* mRNA levels (Fig. 1I, J, and Fig. S1E). An inverse association between melanoma cell pigmentation and EZH2 protein was observed in vivo in PDX melanomas (Fig. 1K).

### EZH2 alters pigmentation and malignant behavior independently of its methyltransferase function

**Fig. 2:**
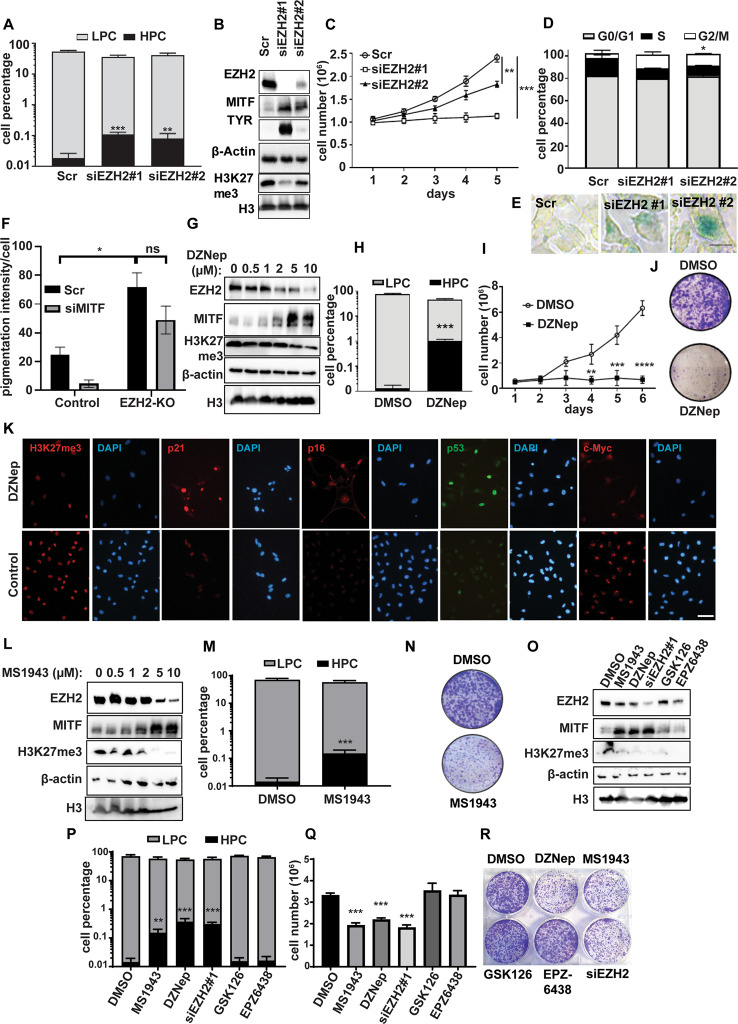
Pharmacological inhibition of EZH2 abundance, but not of its methyltransferase activity, induces pigmented cell phenotypes in melanoma. **A**–**E** B16-F10 murine melanoma cells were transfected with either of two siRNAs against *Ezh2* or scrambled controls, and analysed as follows: **A** HPC and LPC cell percentages assessed by flow cytometry, (**B**) Ezh2, Mitf, Tyr and H3K27me3 protein levels measured by western blot, including β-actin and H3 as loading controls, (**C**) cell growth measured by Trypan blue cell counting over 5 days, (**D**) cell cycle analysis measured by propidium iodide staining, and (**E**) cell senescence determined by β-gal staining (green). **F** Control and EZH2-KO 28:B4:F3 cells transfected with scrambled control or siMITF were stained with Fontana Masson to measure pigmentation. **G**–**K** B16-F10 cells treated with 2 μM DZNep or DMSO (control) for 3 days were analysed for: (**G**) Ezh2, Mitf, and H3K27me3 expression by western blot, (**H**) HPC and LPC cell percentages by flow cytometry, (**I**) cell growth (7 days), (**J**) clonogenicity after low-density seeding (crystal violet stain), and (**K**) H3K27me3, p21, p16, p53, and c-Myc IF staining. Scale bar: 50 µm. **L**–**O** B16-F10 cells were treated with 2 μM MS1943 or DMSO (control) for 3 days prior to: (L) Western blot analysis of Ezh2, Mitf, and H3K27me3 protein levels, (**M**) evaluation of HPC and LPC cell percentages by flow cytometry, (**N**) and clonogenicity after low-density seeding (crystal violet stain). **O**–**R** B16-F10 cells were treated with 2 μM MS1943, 2 μM DZNep, siEzh2 #1, 2 µM GSK126, 2 µM EPZ6438 or DMSO (control) for 3 days prior to: (**O**) Western blot analysis of Ezh2, Tyr, and H3K27me3 expression, (**P**) evaluation of HPC and LPC cell percentages by flow cytometry, (**Q**) calculation of cell numbers counted by Trypan blue, and (**R**) clonogenicity estimation after low-density seeding (crystal violet stain). Clonogenicity was assessed in pre-treated (3 days) cells seeded at 2000 cells in 6-well plate followed by crystal violet staining (0.5% in methanol) after incubation for 10 days in drug-free media. Representative images of *n* = 3 biological replicates are shown for western blots (**B**, **G**, **L**, **O**), clonogenicity plates (**J**, **N**, **R**) and β-gal staining (**E**). Data for **A**, **C**, **D**, **F**, **H**, **I**, **M**, **P** and **Q** were derived from three independent experiments and are presented as means ± SD, analyzed by one-way ANOVA plus Tukey’s multiple comparison test. ns: non-significant. *p < 0.05, **p < 0.01, ***p < 0.001, ****p < 0.0001.

To investigate EZH2 in melanoma cell pigmentation, we suppressed EZH2 protein and/or EZH2 activity in pigmented melanoma cells. Because high EZH2 expression is seen in LPCs, correlations between EZH2 and pigmentation were investigated in B16-F10 cells, which display variable pigmentation. Unsorted B16-F10 cells were EZH2-silenced using two siRNAs, which induced pigmented phenotypes (Fig. 2A) and upregulation of melanocytic differentiation markers MITF and TYR that were proportional to knockdown efficiency (Fig. 2B). siEZH2 also reduced cell viability in a time-dependent manner (Fig. 2C) and caused G2/M cell cycle phase accumulation (Fig. 2D), senescent phenotypes (Fig. 2E), and induction of apoptosis, depending on the degree of EZH2 depletion (Fig. S2A). EZH2 knockout by CRISPR-Cas9 induced MITF in pigmented melanoma cell lines (28:B4:F3 and C006-M1), but not in non-pigmented SK-MEL-28 and A375 cells (Fig. S2B). Interestingly, EZH2 knockout-induced pigmentation was partly, but not significantly, reduced by MITF silencing in 28:B4:F3 cells (Fig. 2F and Fig. S2C, D).

When B16-F10 cells were treated with DZNep, a general methyltransferase inhibitor that also promotes ubiquitin-mediated proteasomal degradation, we observed increased MITF, decreased EZH2 and H3K27me3 (Fig. 2G), and more prominent pigmented cell phenotypes (Fig. S2E, F), including by flow cytometric analysis (Fig. 2H and Fig. S2G). Extracellular melanin was also induced after EZH2 knockdown or DZNep (Fig. S2H).

In line with siRNA experiments, DZNep-treated B16-F10 cells displayed reduced clonogenicity compared to untreated controls (Fig. 2I, J), as well as G2/M phase accumulation indicative of cell cycle arrest (Fig. S2I), and senescent phenotypes (Fig. S2J, K). In addition, upregulation of EZH2-suppressed target genes *Cdkn1a* (p21) and *Cdkn2a* (p16) was noted following DZNep treatment (Fig. 2K). DZNep also increased nuclear p53 and reduced nucleolar c-Myc, consistent with induction of HPC phenotypes upon EZH2 inhibition (Fig. 2K), and reduced LPC markers such as nucleolin localization to nucleoli [42] (Fig. S2L).

We also targeted EZH2 with the EZH2-specific degrader MS1943 [52], which simultaneously reduced EZH2 and H3K27me3, and upregulated MITF (Fig. 2L). Similar to effects of siEZH2 and DZNep, MS1943-treated cells showed higher melanin content (Fig. 2M), greater senescence and reduced clonogenicity (Fig. 2N–R and Fig. S2K).

Although EZH2 methyltransferase inhibitors GSK126 and EPZ6438 fully inhibited EZH2 methyltransferase activity as measured by H3K27me3 (Fig. 2P), they had no or minimal effects on pigmentation, viability or clonogenicity of the melanoma cells we tested (Fig. 2Q–R). Thus, specific inhibition of EZH2 methyltransferase did not phenocopy the interventions that reduced total EZH2 protein. This suggests that EZH2 abundance, but not its methyltransferase activity, promotes the LPC state.

### EZH2 expression is higher in metastases than primary melanomas

**Fig. 3:**
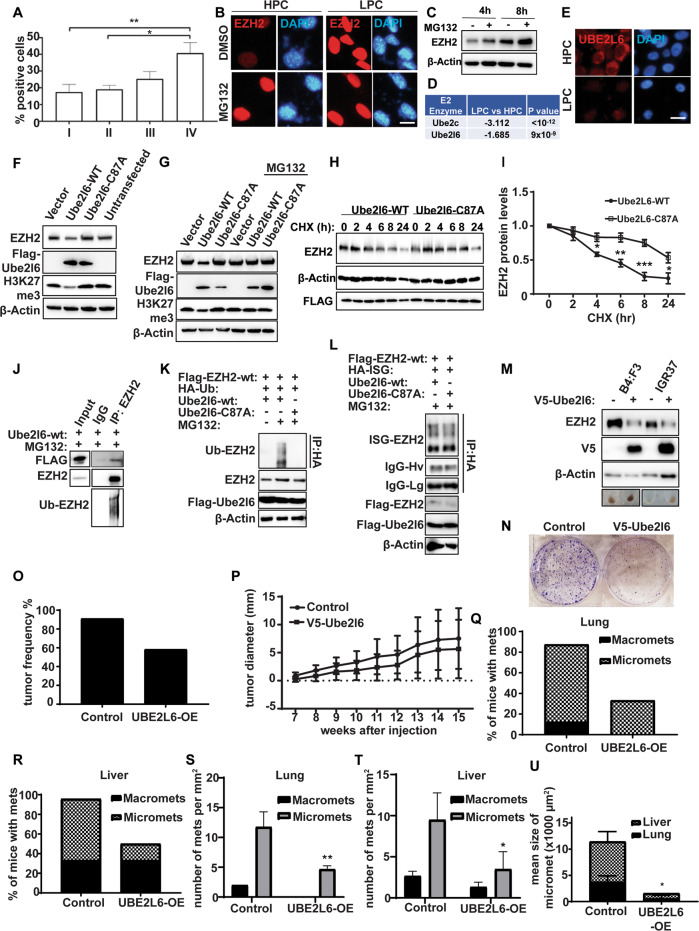
Ezh2 is proteasomally degraded via Ube2l6. **A** EZH2 expression according to melanoma tumor stage in the Melanoma Research Victoria patient cohort. Stage I (*n* = 12 patients), Stage II (*n* = 10 patients), Stage III (*n* = 9 patients) and Stage IV (*n* = 8 patients). Data are presented as mean ± SD and analyzed by one-way ANOVA plus Tukey’s multiple comparison test. *p < 0.05, **p < 0.01. **B** Ezh2 (red) IF in HPCs and LPCs sorted from B16-F10 cells and treated with 10 µM MG132 or DMSO control for 16 h. DAPI (blue): nuclei. Scale bar: 10 µm. **C** Ezh2 protein levels determined by western blot in B16-F10 cells treated with 10 µM MG132 or DMSO control for 4 h or 8 h. **D** E2 ligases downregulated in RNAseq data from LPCs vs HPCs. **E** Ube2l6 IF in LPCs and HPCs from B16-F10 cells. Scale bar: 10 µm. **F** Ezh2 and H3K27me3 levels were determined by western blot in B16-F10 cells transfected with Flag-tagged Ube2l6-WT, Flag-tagged Ube2l6-C87A (enzyme-dead) or empty vector. (**G**) As in (**F**), with or without 10 µM MG132 treatment for 16 h. **H** Stability of endogenous Ezh2 protein determined by western blot in B16-F10 cells transfected with Flag-tagged Ube2l6-WT or Flag-tagged Ube2l6-C87A, followed by 50 µg/mL CHX treatment for the indicated times. **I** Densitometry for western blots shown in (**H**). Data from three independent experiments are presented as mean ± SD and were analyzed by one-way ANOVA plus Tukey’s multiple comparison test. *p < 0.05, **p < 0.01, ***p < 0.001. **J** Flag-tagged Ube2l6-WT was overexpressed in B16-F10 cells maintained in 10 µM MG132 for 16 h. Interactions between endogenous Ezh2 and Ube2l6 were determined by immunoprecipitation with anti-Ezh2 antibody followed by western blotting with anti-Flag antibody. **K** HEK293 cells were co-transfected with Flag-tagged EZH2 and Flag-tagged Ube2l6-WT or Flag-tagged Ube2l6-C87A, together with HA-tagged ubiquitin, or (**L**) HA-tagged ISG15 in the presence of 10 µM MG132. The ubiquitination (**K**) and ISGylation (**L**) of EZH2 were determined by anti-HA IP followed by western blot with anti-EZH2 antibody. **M** 28:B4:F3 and IGR37 cells were infected with V5-tagged empty vector or V5-tagged UBE2L6 lentiviral particles. Positive clones were selected by incubation in 2 µg/µL puromycin for 2 weeks. EZH2 was detected by western blot in stably transfected cells. Representative cell pellets are shown (bottom row). **N** Clonogenicity of 28:B4:F3 cells assessed by CV staining. **O** Tumor engraftment in NSG mice harboring control or V5-UBE2l6-WT vector cells 15 weeks after injection. **P** Volumes of tumors in NSG mice injected with 28:B4:F3 cells harboring control or V5-UBE2l6-WT vector after 15 weeks. The percentages of mice with macro- and micro-metastasis, and number of metastases per mm^2^ to either (**Q**, **S**) lung, or (**R**, **T**) liver, respectively. **U** Mean area of micro-metastases in lungs and liver. Control mice number = 11, UBE2L6-OE mice number = 11. Data analyzed by student t-test. *p < 0.05.

If EZH2 positively regulates malignant behaviors in melanoma cells, then its expression might be predicted to increase during disease progression. As increasing EZH2 protein expression was observed from benign nevi to metastatic melanoma [7, 16, 17] and as melanoma patient survival was inversely correlated with EZH2 expression [7], we sought to extend these findings. Analysis of TCGA melanoma samples showed no correlation between *EZH2* mRNA levels and overall patient survival or disease staging in melanoma (Fig. S3A, B), consistent with our findings (Fig. 1I, J and Fig. S1E). However, in 39 melanoma samples obtained from patients in the Melanoma Research Victoria cohort, incrementally increasing EZH2 protein expression was observed from early stage to metastatic disease (Fig. 3A), confirming published data [7].

### UBE2L6 promotes ubiquitin-associated proteosomal degradation of EZH2 in melanoma cells

To understand discrepancies between *EZH2* mRNA and EZH2 protein levels in melanoma cells, we investigated post-translational mechanisms that might regulate EZH2. Since ubiquitin-mediated proteasomal degradation plays a pivotal role in protein abundance by affecting protein stability [53], we assessed EZH2 levels pre- and post-treatment with a proteasome inhibitor, MG132, in sorted LPCs and HPCs, and in unsorted B16-F10 cells. MG132 treatment increased EZH2 protein in HPCs, but not in LPCs, in a time-dependent manner (Fig. 3B, C), suggesting increased proteasomal degradation of EZH2 in HPCs compared to LPCs.

To elucidate mechanisms underlying this, we interrogated our RNAseq data to identify candidate ubiquitin pathway enzymes involved in the post-translational regulation of EZH2. Among E1 and E2 enzymes, *Uba7* (Fig. S3C), and *Ube2l6* and *Ube2c* (Fig. 3D, Fig., S3D), respectively, were significantly decreased in LPCs compared to HPCs. To explore ubiquitination in EZH2 regulation, we focused on Ube2l6, as Ube2c overexpression had trivial effects on EZH2 protein (Fig. S3E), and E1 enzymes have a wide range of substrates.

We first confirmed lower expression of Ube2l6 in LPCs compared to HPCs sorted from B16-F10 (Fig. 3E). In gene targeting experiments, Ube2l6-WT overexpression in B16-F10 LPCs downregulated Ezh2 protein (Fig. 3F and Fig. S3F), while cells overexpressing enzyme-dead Ube2l6-C87A displayed increased Ezh2 (Fig. 3F and Fig. S3F). The Ube2l6-mediated decrease in Ezh2 protein was reversed by MG132, highlighting the critical role of proteasomal degradation in regulation of Ezh2 (Fig. 3G). Inhibitory effects of Ube2l6 on Ezh2 protein were verified by Ube2l6 knock-down with siUbe2l6-3’-UTR, which increased Ezh2 expression (Fig. S3G).

We next investigated Ezh2 protein stability and interactions. Following cycloheximide treatment, which inhibits protein synthesis, EZH2’s half-life was ~5 h in Ube2l6-WT overexpressed cells and > 24 h in Ube2l6-C87A cells lacking ligase activity (Fig. 3H, I), consistent with a role for Ube2l6 in Ezh2 degradation. Ezh2-Ube2l6 protein-protein interactions were verified by immunoblotting of EZH2 immunoprecipitates from Flag-tagged Ube2l6-overexpressing HEK293 cells treated with MG132 (Fig. 3J).

The presence of higher-than-expected molecular weight EZH2 bands in western blots (Fig. 3J, lowest panel) prompted us to investigate ubiquitination of EZH2, finding that EZH2 was ubiquitinated by UBE2L6-WT, but not by enzyme-dead UBE2L6-C87A, in both HEK293 and B16-F10 cell lines (Fig. 3K and Fig. S3H). Although UBE2L6 is a critical enzyme in ISGylation, it had no effect on EZH2 ISGylation (Fig. 3L). DZNep reduced EZH2 expression in B16-F10 cells and upregulated *Ube2l6* mRNA (Fig. S3I, Table S6), and Ube2l6 knockdown reversed DZNep-induced EZH2 degradation, TYR expression and cell viability (Fig. S3I). These data show that UBE2L6 acts post-translationally to reduce EZH2 abundance through ubiquitin-associated proteasomal degradation.

### UBE2L6 reduces EZH2 abundance, tumorigenicity and metastasis in melanoma

To evaluate functional consequences of UBE2L6-EZH2 interactions in melanoma, we developed stable UBE2L6-WT-overexpressing pigmented 28:B4:F3 and IGR37 cells. Overexpression of UBE2L6-WT decreased EZH2 protein and cell viability, clonogenicity, invasion and pigmentation (Fig. 3M, N and Fig. S3J, K). Following subcutaneous injection into mice, tumor formation was impeded in UBE2L6-overexpressing 28:B4:F3 recipient mice compared to controls (Fig. 3O), although differences in tumor volume were not statistically significant at 15 weeks (Fig. 3P). While control tumors were partially pigmented, they were all pigmented in UBE2L6-overexpressed tumors (Fig. S4A), which also demonstrated lower EZH2 protein levels (Fig. S4B) and seeded fewer metastases (Fig. 3Q–T) that were smaller and also more pigmented than metastases from control tumors (Fig. 3U and Fig. S4C–E). Interestingly, all tumor bearing mice developed lymph node metastasis irrespective of UBE2L6 status (data not shown).

**Fig. 4:**
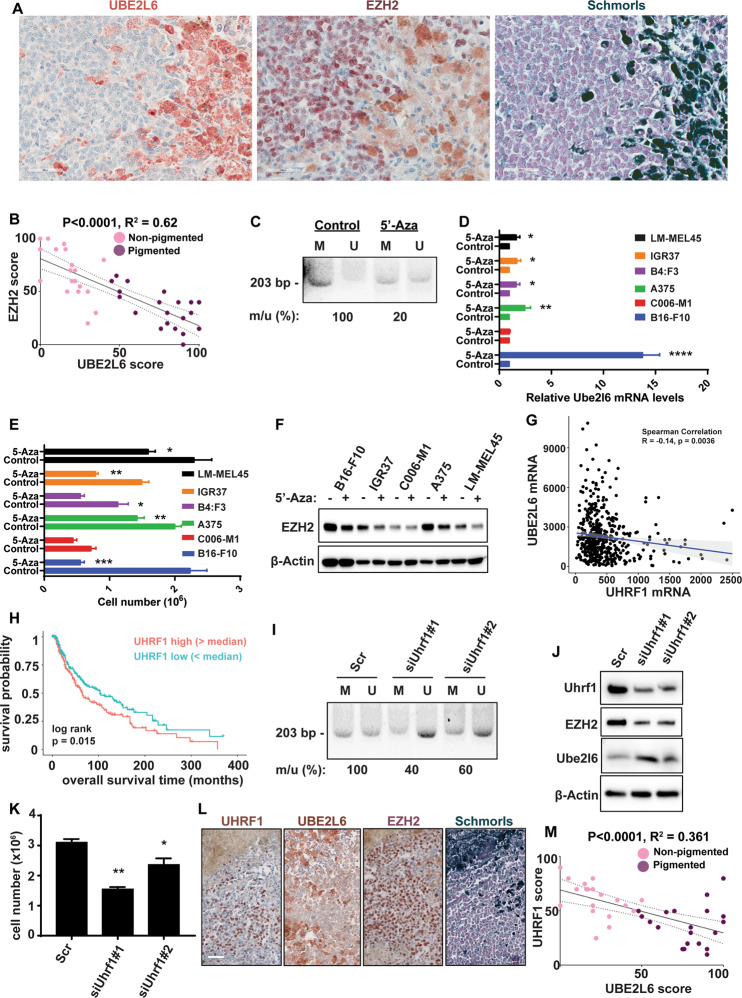
UBE2L6 promoter methylation via UHRF1 depletes UBE2L6 and stabilizes EZH2. **A** Immunohistochemical staining of EZH2 and UBE2L6 in representative Schmorl’s stained pigmented human melanomas. Scale bar: 50 μm. **B** UBE2L6 protein scores (x-axis) negatively correlated with EZH2 protein scores (y-axis) in patient melanomas. Pink dots correspond to non-pigmented and purple dots to pigmented patient samples. *p*-value calculated from a linear regression analysis. *R* = correlation coefficient. Protein score = the percentage of immunopositive cells × immunostaining intensity. **C**–**F** Indicated cell lines were treated with 2 µM 5’-Azacitidine or DMSO (vehicle) for 72 h prior to: (**C**) methylation specific primer (MSP) analysis of methylation at the UBE2L6 promoter (M: Methylated specific primer; U: Unmethylated specific primer; number below image represents percent ratio of methylated (m) to unmethylated (u) DNA quantified by ImageJ after normalization to controls (m/u), (**D)** *UBE2L6* mRNA quantification with RT-qPCR, (**E**) cell number by Trypan blue cell counting, and (**F**) EZH2 protein quantification with western blot. **G** Correlation between *UBE2L6* and *UHRF1* mRNA levels in TCGA cutaneous melanoma samples. **H** Kaplan-Meier curves of overall survival of TCGA cutaneous melanoma patients (*n* = 427) stratified by *UHRF1* mRNA levels. *p* = 0.015 (log-rank test). **I**–**K** A375 human melanoma cells were transfected with one of two siRNAs against *Uhrf1,* or scrambled controls, followed by: (**I**) MSP analysis of *UBE2L6* promoter as in (**C**), (**J**) EZH2 and UBE2L6 protein estimation by western blot, and (**K**) cell number by Trypan blue cell counting. **L** Immunohistochemical staining of UHRF1 and UBE2L6 in representative Schmorl’s stained pigmented human melanomas. Scale bar, 50 μm. **M** UBE2L6 protein scores (x-axis) in melanoma samples negatively correlated with UHRF1 scores (y-axis) in individual patients. Pink dots correspond to non-pigmented and purple dots to pigmented patient samples. *P*-value calculated from linear regression analysis. R = correlation coefficient. Protein score = percentage of immunopositive cells × immunostaining intensity. Data for **D**, **E** and **K** from three independent experiments are presented as means ± SD, analyzed by one-way ANOVA plus Tukey’s multiple comparison test. *p < 0.05, **p < 0.01, ***p < 0.001, ****p < 0.0001.

Further to examine UBE2L6 in human melanoma, we performed immunohistochemical (IHC) staining on 19 pigmented and 20 non-pigmented human melanomas. Pigmented regions in tumors exhibited high UBE2L6 but low EZH2 expression, whereas non-pigmented regions displayed the inverse phenotype (Fig. 4A and Fig. S5A). To complement these observations, we also analyzed UBE2L6 expression in five random fields of tumor sections and plotted it against EZH2 expression scores, revealing a negative correlation between UBE2L6 and EZH2 (linear regression *R*^*2*^ = 0.62, *p* < 0.0001; Fig. 4B). Interestingly, a mild decline (*p* = 0.0575) was noted in cytosolic UBE2L6 from early stage to metastatic melanoma, inversely correlated with EZH2 (Fig. S5B and Fig. 3A). We also analyzed TCGA melanoma data, finding a correlation between low *UBE2L6* mRNA expression and poor melanoma survival (*p* < 0.0001, Fig. S5C). Interestingly, *UBE2L6* mRNA was negatively correlated with its promoter CpG methylation levels (Spearman correlation *r* = −0.63, *p* = 2.2 × 10^−16^, Fig. S5D). These data implicate UBE2L6 as a tumor suppressor in melanoma.

### Promoter methylation via UHRF1 suppresses UBE2L6 and upregulates EZH2 in melanoma

We next evaluated promoter methylation as a potential mechanism of UBE2L6 regulation. 5-Aza-2′-deoxycytidine (5’-Aza) was used to inhibit DNA methyltransferases and relieve CpG methylation in A375 cells, followed by methylation-sensitive PCR. 5’-Aza induced an 80% decline in *UBE2L6* CpG island methylation spanning −582 to −378 from the transcription start site (Fig. S5E, Fig. 4C), increased UBE2L6, reduced EZH2, and impaired viability in multiple melanoma cell lines (Fig. 4D–F).

To understand UBE2L6 methylation, we focused on the RING E3 ubiquitin ligase UHRF1, previously shown to downregulate UBE2L6 [54] and promote melanoma cell proliferation [55]. Initial analysis of TCGA melanoma data revealed that *UHRF1* expression inversely correlated with *UBE2L6* (Spearman correlation *r* = −0.14, *p* = 0.0036 Fig. 4G) and melanoma survival (*p* = 0.015, Fig. 4H). We thus tested whether UHRF1 could regulate *UBE2L6* promoter methylation in vitro. UHRF1 silencing in A375 cells resulted in a 40–60% decline in *UBE2L6* promoter methylation (depending on silencing efficiency) (Fig. 4I), increased UBE2L6, decreased EZH2 (Fig. 4J. and Fig. S5F), and reduced viability in melanoma cell lines (Fig. 4K and Fig. S5G). IHC staining of UHRF1 expression in human melanomas showed that highly pigmented cells exhibiting increased UBE2L6 and decreased EZH2 also had low UHRF1 expression (Fig. 4L–M). These data link *UBE2L6* promoter methylation via UHRF1 to UBE2L6 suppression and EZH2 stabilization in melanoma cells.

### The E3 ligase UBR4 interacts with UBE2L6 and EZH2

**Fig. 5:**
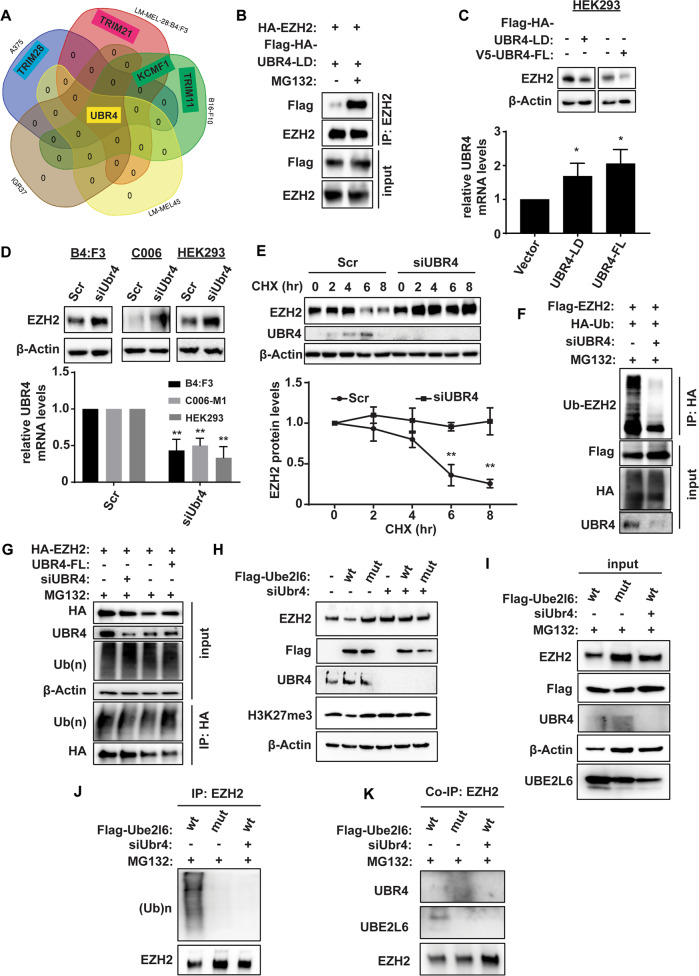
UBR4 is the E3 ligase of EZH2. **A** Venn diagram depicting overlap of proteins co-immunoprecipitating with EZH2 from C006-M1, 28:B4:F3, IGR37, A375 and LM-MEL-45 cells (data derived from *n* = 3 biological replicates). **B** HA-tagged EZH2 and Flag-HA-tagged UBR4-LD (ligase domain) were co-expressed in HEK293 cells maintained ±MG132. Interactions between EZH2 and UBR4-LD were determined by immunoprecipitation with anti-EZH2 antibody and western blotting with anti-Flag antibody. **C** EZH2 protein was determined in HEK293 cells overexpressing either UBR4-LD or UBR4-FL (full length). *UBR4* overexpression was measured by qPCR (lower panel: quantification relative to vector control, error bars: + SD). **D** UBR4 in 28:B4:F3, C006-M1 and HEK293 cells was knocked down by siRNA. EZH2 levels determined by western blot (top panels). Knockdown efficiency of *UBR4* measured by qPCR as in (**C**). **E** Stability of EZH2 according to UBR4 levels (unaltered vs knockdown) was determined by western blot in A375 cells treated with cyclohexamide (CHX). Representative blot shown at the top, time-course plot at the bottom. **F** Ubiquitination of EZH2 was determined by anti-HA IP followed by western blot in A375 cells. **G** HEK293 cells with or without *siUBR4* were transfected with HA-tagged EZH2 or V5-tagged UBR4-FL followed by 50 µM MG132 treatment for 4 h. Ubiquitination of EZH2 was determined by western blot with anti-ubiquitin (Ub) antibody. **H** B16-F10 cells ±*siUBR4* were transfected with either Flag-tagged *Ube2l6-WT* (“wt”) or Flag-tagged *Ube2l6-C87A* (“mut”) and EZH2 levels determined by western blot. **I**–**K** To evaluate EZH2/UBR4/UBE2L6 interactions, B16-F10 cells ±*siUBR4* were transfected with either Flag-tagged *Ube2l6-WT* (“wt”) or Flag-tagged *Ube2l6-C87A* (“mut”) followed by 50 µM MG132 treatment for 4 h and then: (**I**) EZH2, UBR4 and UBE2L6 protein estimation by western blot, (**J**) determination of ubiquitination of EZH2 by western blot with anti-Ub antibody, and (**K**) co-immunoprecipitation of UBR4 and UBE2L6 with EZH2 antibody. Data represent *n* = 3 biological replicates. Data for **D** and **E** are from three independent experiments and presented as means ± SD, analyzed by one-way ANOVA plus Tukey’s multiple comparison test. **p < 0.01.

The activity of E2 conjugating enzymes relies on their cooperation with E3 ligases through which occur direct interactions with substrates. To identify EZH2-bound E3 ligases in melanoma, EZH2-co-immunoprecipitated lysates obtained from a variety of melanoma cell lines (A375, 28:B4:F3, B16-F10, LM-MEL45 and IGR37) were subjected to Liquid Chromatography-Mass Spectrometry (LC-MS). Besides PRC1 and PRC2 complex proteins, we identified novel EZH2 binding proteins (Table S8). We focused on UBR4, because it was the only common EZH2-interacting E3 ligase identified in the cell lines tested (Fig. 5A). No differences in UBR4 expression were seen between HPCs and LPCs (Fig. S5H) and pigmented patient samples (Fig. S5I), in contrast to UBE2L6 (Fig. 3E).

To confirm UBR4 as an EZH2 E3 ligase, interactions between EZH2 and UBR4-LD (ligase domain) were assessed by CoIP-coupled western blotting. EZH2 interacted with the E3 ligase domain of UBR4 in a manner that was enhanced by MG132 (Fig. 5B and Fig. S5J), and was diminished by UBR4-LD and UBR4-FL (full length) overexpression (Fig. 5C). As expected, UBR4 knockdown in 28:B4:F3, C006 and HEK293 cells increased EZH2 expression (Fig. 5D) and stability (Fig. 5E) via decreased EZH2 ubiquitination (Fig. 5F). UBR4-FL overexpression, on the other hand, increased EZH2 ubiquitination (Fig. 5G). These data indicate that UBR4 is an E3 ligase of EZH2.

To test cooperation between UBE2L6, UBR4 and EZH2, Ube2l6-WT- or Ube2l6-C87A-overexpressing B16-F10 cells were subjected to Ubr4 silencing, which reversed Ube2l6-WT-mediated Ezh2 degradation (Fig. 5H, I) and ubiquitination in B16-F10 cells (Fig. 5J). Furthermore, interactions between Ubr4 and Ezh2 were demonstrated by Ube2l6-WT overexpression, but not by enzyme dead Ube2l6-C87A (Fig. 5K). More importantly, UBE2L6-EZH2 interactions were ablated by UBR4 silencing (Fig. 5K). These data support a UBE2L6-UBR4 interaction with EZH2 to facilitate EZH2 ubiquitination in melanoma cells.

### EZH2 K381 residues are sites of UBE2L6-UBR4-mediated ubiquitination

**Fig. 6:**
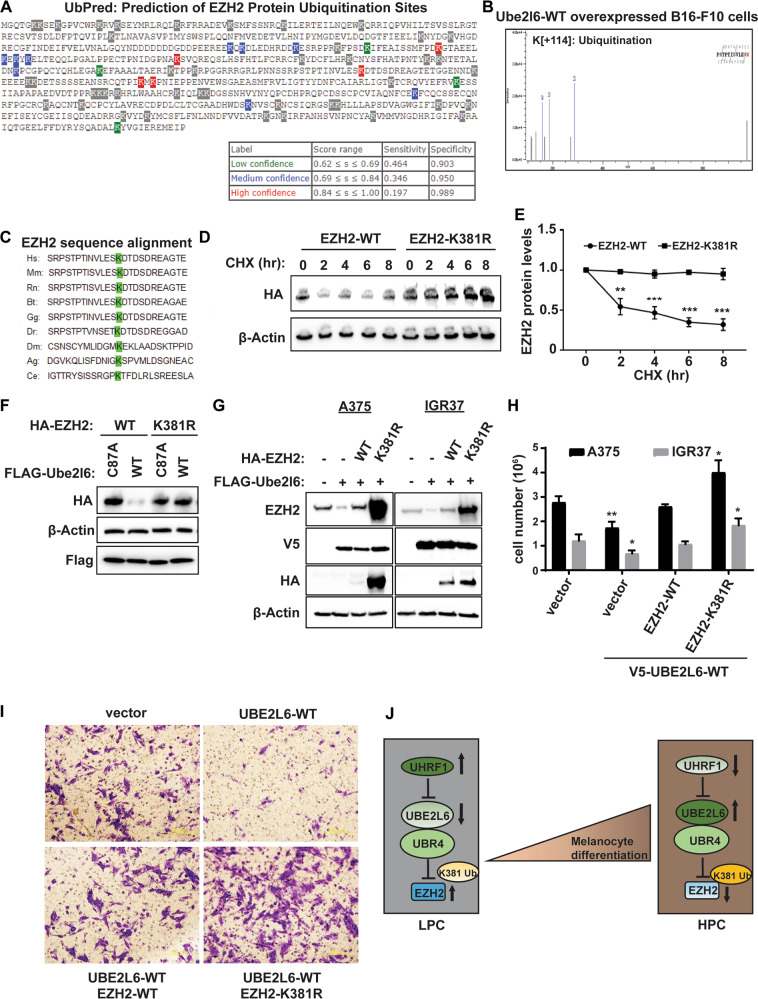
The K381 residue on EZH2 is ubiquitinated by UBE2L6/UBR4 in melanoma cells. **A** Prediction of ubiquitination sites on human EZH2 was made using UbPred, shown as low (green), medium (blue) and high (red) confidence lysine ubiquitination sites (www.ubpred.org) (**B**) LC-MS analysis of mouse Ezh2 K376 ubiquitination in B16-F10 cells. **C** Sequence alignment of residues 368–392 of human (Hs) EZH2 protein against Mus musculus (Mm, mouse), Rattus norvegicus (Rn, rat), Bos taurus (Bt, cow), Gallus gallus (Gg, chicken), Danio rerio (Dr, zebrafish), Drosophila melanogaster (Dm, fruit fly), Anopheles gambiae (Ag, mosquito), and Caenorhabditis elegans (Ce, worm) showing conservation of K381 residues. **D** HEK293 cells with HA-tagged EH2-WT or HA-tagged EZH2-K381A were treated with 50 µg/mL CHX (cycloheximide) for the durations indicated. Stability of HA-tagged EZH2 protein was determined by western blot with anti-HA antibody. **E** Quantitated time course of EZH2 protein levels from **(D)**. **F** B16-F10 cells were transfected with either Flag-tagged *Ube2l6-WT* or Flag-tagged *Ube2l6-C87A* (mutant) and HA-tagged *EZH2-WT* or HA-tagged *EZH2-K381A*, and HA-EZH2 levels determined by western blot with anti-HA antibody. **G** A375 and IGR37 cells transduced with *V5-UBE2L6-WT* were transfected transiently with either HA-tagged *EZH2-WT* or HA-tagged *EZH2-K381R*. Endogenous EZH2 levels were determined 48 h post-transfection with EZH2 antibody, HA-EZH2 levels were determined with anti-HA antibody and V5-UBE2L6 levels with V5 antibody. **H** Cells treated as in (**G**) were counted by Trypan blue 48 h post-transfection. **I** Invasion of cells treated as in (**G**) was measured 72 h post-transfection using Boyden chamber matrigel invasion assays followed by crystal violet staining. **J** Proposed model for UHRF1/UBE2L6/UBR4-mediated regulation of EZH2 and thereby melanocytic differentiation phenotypes in melanoma. Data for **E** and **H** from three independent experiments are presented as means ± SD, analyzed by one-way ANOVA plus Tukey’s multiple comparison test. *p < 0.05, **p < 0.01, ***p < 0.001.

We next aimed to determine ubiquitination sites on EZH2, predicted by UbPred [56] to be at EZH2 K381 in humans and K376 in mice (Fig. 6A). As endogenously ubiquitinated EZH2 was undetectable by LC-MS in all tested melanoma cells (data not shown), we assessed Ube2l6-WT overexpressing B16-F10 cells, detecting Ezh2 ubiquitination at residue K376 (Fig. 6B). As expected, LC-MS could not detect EZH2 ubiquitination upon enzyme dead Ube2l6-C87A overexpression or Ubr4 silencing.

To complement these data, we performed sequence alignment, revealing that the K381 residue has been conserved in evolution, suggesting its critical for the K381 in EZH2 regulation (Fig. 6C). To test EZH2 K381 ubiquitination functionally, EZH2 K381R mutant proteins were expressed in HEK293 cells, inducing EZH2 stability (Fig. 6D, E) and resistance to UBE2L6-mediated EZH2 degradation (Fig. 6F). Accordingly, melanoma cell viability and invasiveness were promoted by overexpression of EZH2-K381R in UBE2L6-overexpressing cells (Fig. 6G–I). These data implicate UBE2L6 as a tumor suppressor in melanoma that couples with UBR4 to direct ubiquitination of EZH2 K381 residues and ubiquitin-mediated degradation of EZH2 (Fig. 6J).

## Discussion

We identified EZH2 as a master regulator of melanoma ITH and promoter of malignant behavior in melanoma cells. Genetic or pharmacological depletion of EZH2, but not specific inhibition of its methyltransferase activity, inhibited melanoma cell tumorigenicity and invasion, indicating a critical methyltransferase-independent function for EZH2 in melanoma. This effect was modulated by UHRF1/UBE2L6/UBR4-mediated inhibition of EZH2 through proteosomal degradation, implicating UHRF1 and UBE2L6 as regulators of ITH and disease propagation in melanoma. Targeting these enzymes might be exploitable in anti-melanoma therapy.

Previous studies implicated genes involved in melanin biosynthesis and melanocytic differentiation as key targets of EZH2-directed histone methylation [57, 58], leading to altered pigmentation and differentiation phenotypes in melanoma cells [59]. Indeed, we found that EZH2 protein was inversely associated with pigmentation in both melanoma cells and patient melanomas. However, unlike other studies, we found that selective EZH2 methyltransferase inhibition using GSK126 or EPZ6438 did not substantially affect melanocytic gene expression [58] or cell pigmentation. This discrepancy may be explained by off-target effects of high inhibitor doses used in other studies.

It is thought that phenotype switching produces melanoma cell populations of varied proliferative and invasive activity. Invasive melanoma cells have lower pigment levels compared with non-invasive cells [4, 56, 59, 60] and Ezh2 promotes the low-pigment phenotype in vivo by suppressing Oca2, among other targets [59]. Although Pinner et al., [60] found that non-pigmented invasive melanoma cells metastasize to lung and lymph nodes and reverted to pigmented phenotypes after colonization, we did not observe this in our study. Rather, we found that melanoma cells with high EZH2 expression generally formed partially pigmented tumors following subcutaneous implantation, with non-pigmented metastatic foci in the liver, and that UBE2L6 over-expressing melanoma cells with low EZH2 formed highly pigmented tumors with either no or only small and pigmented metastatic foci.

It was also suggested that EZH2 is required for efficient lung and lymph node colonization by metastatic melanoma [58, 61]. Consistent with this, we found that metastases to the lung and liver were reduced from tumors formed by UBE2L6-overexpressing cells with suppressed EZH2. Interestingly, all tumor-bearing mice developed lymph node metastasis regardless of UBE2L6 status. This suggests that EZH2 may have a greater role in facilitating hematogenous metastasis rather than early dissemination into lymphatics. Alternatively, other UBE2L6 targets may regulate melanoma metastasis.

Tumor-suppressive and anti-metastatic roles of UBE2L6 in vivo have not been described, and we found are mediated in melanoma by the ubiquitin-conjugating function of UBE2L6, working cooperatively with the E3 ligase UBR4, to induce EZH2 ubiquitination and protein degradation [53]. We found that UBE2L6 abundance is itself regulated by UHRF1, which induces suppressive *UBE2L6* promoter methylation and supports oncogenic EZH2 proteins. UHRF1 acts as an oncogene in various cancers, including melanoma [54, 55], and downregulates UBE2L6 in cervical cancer [54]. Our studies extend understanding of this oncoprotein by demonstrating UHRF1/UBE2L6/UBR4-mediated EZH2 promotion to enhance LPC phenotypes and disease progression in melanoma, providing a mechanism as to how higher levels of EZH2 are associated with late-stage disease.

Most anti-cancer strategies focused on EZH2 targeting have assumed that EZH2 methyltransferase activity is the key oncogenic activity of this protein. However, oncogenic and methyltransferase-independent functions of EZH2 have been described [26–28, 62], leading to strategies that reduce EZH2 abundance. For example, in breast cancer, DZNep reduces EZH2 protein stability by upregulating the E3 ligase PRAJA1 [35], and MS1943 depletes EZH2 and induces cytotoxicity [52]. Although we found that DZNep reduces EZH2 in melanoma by inducing UBE2L6 expression, its short half-life [63, 64] and broad effects on ubiquitination hinder clinical application. Targeted depletion of EZH2 through facilitating UHRF1/UBE2L6/UBR4-mediated ubiquitination of its K381 residue offers a specific and appealing alternative.

## Supplementary information

Supplementary Figures and Legends

Cell line, chemicals, plasmids, oligos, primers and antibody list

B16-F10 scr vs siEzh2 DEGs GO and GSEA analysis

B16-F10 HPC vs LPC DEGs GO and GSEA analysis

Ezh2 activated genes over-representation analysis (ORA)

Ezh2 repressed genes over-representation analysis (ORA)

- Genes significantly down- and up-regulated in siEZH2 cells compared to scramble B16-F10 cells

EZH2 coexpressed genes in cutaneous melanoma patient TCGA database

LC-MS of EZH2 coimmunoprecipitates from melanoma cells lines

## Data Availability

All relevant data are available from the authors upon request.
