# Supplementary material for: UHRF1/UBE2L6/UBR4-mediated ubiquitination regulates EZH2 abundance and thereby melanocytic differentiation phenotypes in melanoma
Source: Oncogene. 2023 Mar 11;42(17):1360–73. doi: 10.1038/s41388-023-02631-8 (PMC10121471; doi:10.1038/s41388-023-02631-8)
Supplement: Supplementary file 1 — Supplementary Figures and Legends [file 41388_2023_2631_MOESM1_ESM.docx]

**Supplementary Figure and Figure Legends**

**Supplementary Figure 1.**


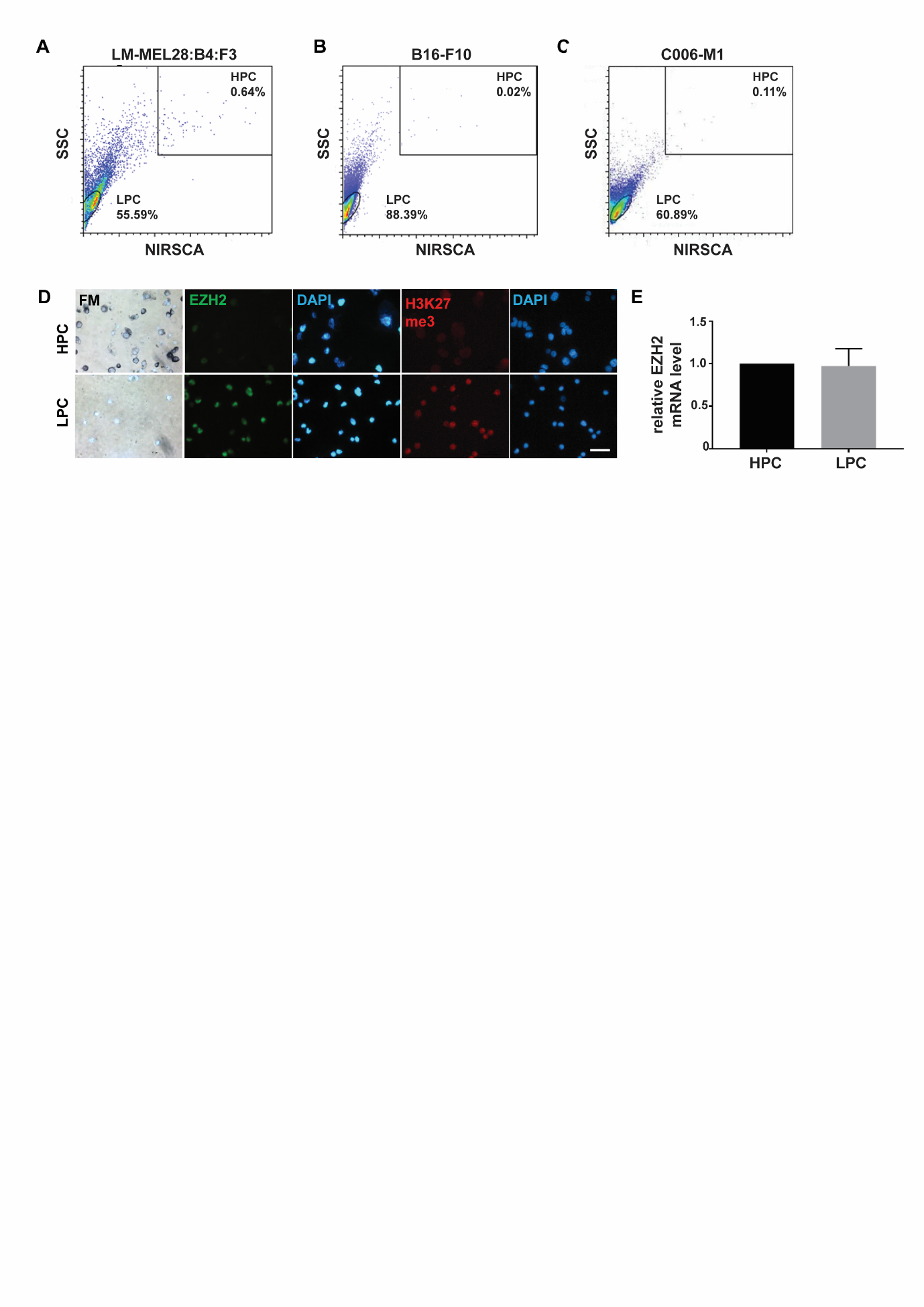


**Supplementary Figure 1.** **EZH2 protein, but not mRNA level is upregulated in LPCs from C006-M1 cells**. SSCA vs NIRSCA FACS analysis of 28:B4:F3 (A), B16-F10 (B) and C006-M1 (C) cells, (D) Bright-field (BF) microscope imaging of Fontana-Masson staining (left panel) and immunofluorescence (IF) images probed for EZH2 (green) and H3K27me3 (red) in HPCs and LPCs from C006-M1 cells. Nuclei shown by DAPI (blue). Scale bar: 50µm. (E) *EZH2* qRT-PCR of HPC and LPCs from C006-M1 cells. n=3 biological replicates.

**Supplementary Figure 2.**

**
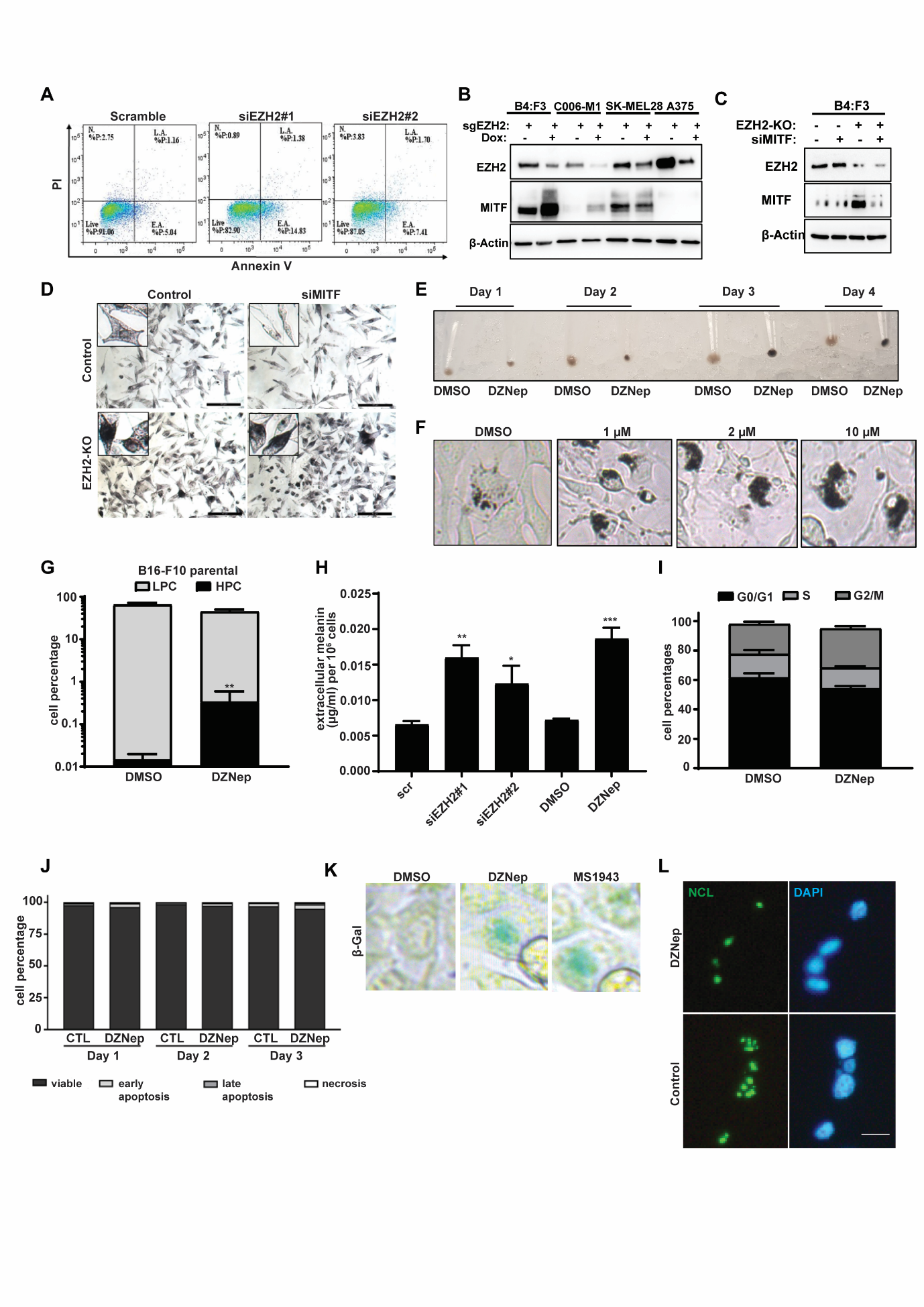
**

**Supplementary Figure 2. Manipulation of EZH2 protein by DZNep or knockout induces HPC phenotypes.** (A) AnnexinV/PI apoptosis analysis in B16-F10 cells transfected with *EZH2* siRNA#1, *EZH2* siRNA#2, or scramble control for 3 days. (B) Western blot analysis of EZH2 and MITF in control and EZH2-knockout 28:B4:F3, C006-M1, SK-MEL28 and A375 cells. (C) Western blot analysis of EZH2, and MITF and (D) Fontana Masson staining in control and EZH2-knockout 28:B4:F3 cells transfected with scrambled control or *siMITF*. (E) Cell pellet imaging of B16-F10 cells treated with DMSO or 2 µM DZNep for the indicated times. (F) Bright-field microscopy of B16-F10 cells treated with DMSO or DZNep for 3 days at differerent doses. (G) HPC and LPC cell percentages in B16-F10 cells treated with DMSO or 2 µM DZNep for 2 days. (H) Extracellular melanin levels determined by melanin assay in B16-F10 cells transfected with scramble control, *siEZH2#1*, or *siEZH2#2*, or treated with DMSO or 2 µM DZNep for 3 days. (I) Cell cycle analysis by PI in B16-F10 cells treated with DMSO or 2 µM DZNep for 3 days, and (J) AnnexinV/PI analysis for the indicated times, (K) cell senescence by β-gal staining in B16-F10 cells treated with DMSO, 2 µM DZNep or 2 µM MS1943 for 3 days. (L) NCL staining in B16-F10 cells treated with DMSO or 2 µM DZNep for 3 days. Scale bar: 10µm. Data for G, H, I and J from three independent experiments are presented as mean ±SD, analyzed by one-way ANOVA plus Tukey’s multiple comparison test. * p<0.05, ** p<0.01, *** p<0.001.

**Supplementary Figure 3.**

**
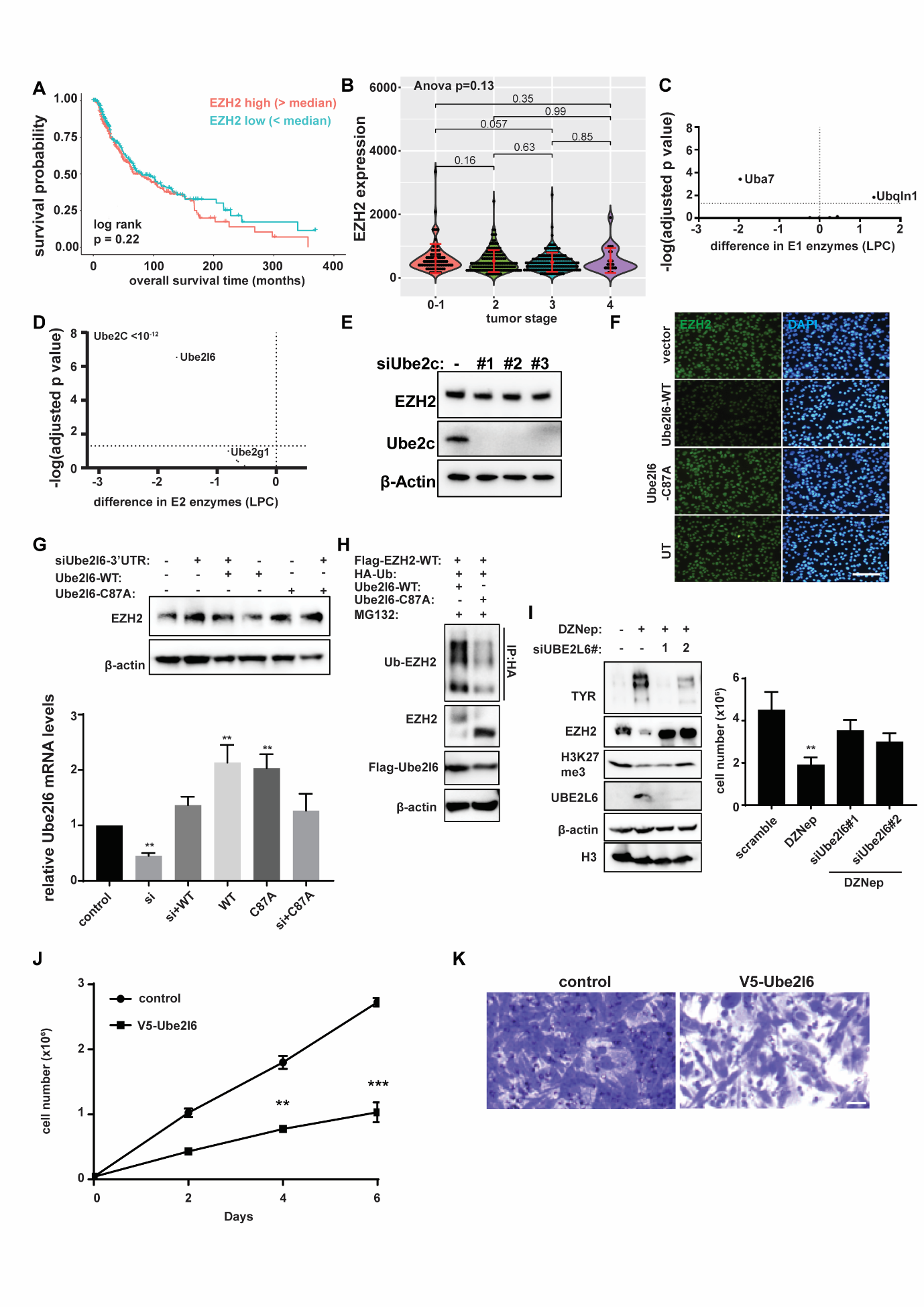
**

**Supplementary Figure 3. EZH2 protein, but not mRNA correlates with melanoma survival and staging, and Ube2l6 is the E2 enzyme of EZH2.** (A) Kaplan-Meier survival (p=0.22 log-rank test) and (B) tumor staging analysis of TCGA cutaneous melanoma patients (n = 427 patients), stratified by EZH2 mRNA levels. (B) EZH2 protein expression according to clinical disease staging in 39 patients from the the Melanoma Research Victoria cohort. Protein score = the percentage of immune-positive cells. Stage I (n=12 patients), Stage II (n=10 patients), Stage III (n=9 patients) and Stage IV (n=8 patients). (C-D) Candidate gene expression screen of (C) E1 ubiquitination genes and (D) E2 ubiquitination genes based on RNAseq data from sorted LPCs and HPCs from B16-F10 cells. (E) Western blot analysis of EZH2 and Ube2c in B16-F10 cells transfected with scramble or three different siUbe2c oligos. (F) Ezh2 immunostaining in B16-F10 cells transfected with empty vector, Flag-tagged Ube2l6-WT, Flag-tagged Ube2l6-C87A (enzyme-dead) or untransfected (UT). Scale bar: 200 µm. (G) Endogenous Ezh2 protein measured by western blot in B16-F10 cells transfected with Ube2l6-WT or Ube2l6-C87A ±siUbe2l6-3’UTR oligos. (H) B16-F10 cells were co-transfected with Flag-tagged EZH2 and Flag-tagged Ube2l6-WT or Flag-tagged Ube2l6-C87A together with HA-tagged ubiquitin in the presence of 10 µM MG132. Ubiquitination of EZH2 was determined by anti-HA IP followed by western blot with anti-EZH2 antibody. (I) Western blot analysis and cell viability assay of EZH2 and TYR in B16 cells with or without two different siUbe2l6 oligos and treated with 2 μM DZNep for 72h. (J) Cells were counted second-daily for 6 days by trypan blue. (K) Boyden chamber invasion assay were performed in 28:B4:F3 cells that were infected with V5-tagged empty vector or V5-tagged UBE2L6 lentiviral particles. Scale bar = 200 µm. Data for I and J from three independent experiments are presented as mean ±SD, analyzed by one-way ANOVA plus Tukey’s multiple comparison test. ** p<0.01, *** p<0.001.

**Supplementary Figure 4.**

**
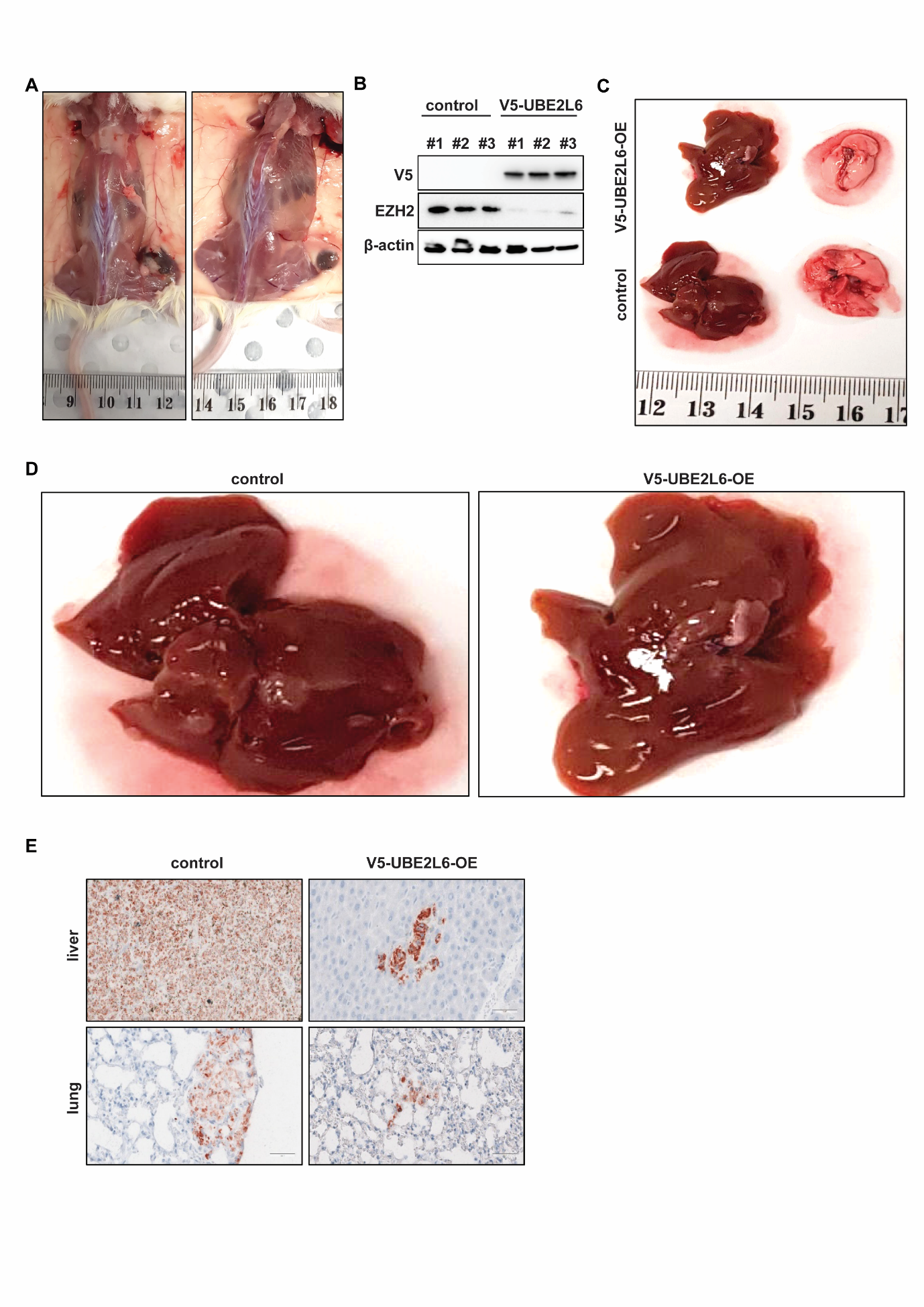
**

**Supplementary Figure 4. UBE2L6 overexpression decreases lung and liver metastasis rates *in vivo*.** (A) Representative images of tumors measured in Fig 3O. (B) Western blot of three representative tumors with anti-V5 and anti-EZH2 antibody. (C) Representative images of liver and lung from mice injected with 28:B4:F3 harboring control or V5-UBE2l6-WT vector. (D) Higher magnification images of livers shown in (C). (E) Representative human mitochondria immunohistochemistry images (brown) of lung and liver sections from mice that harbored a xenografted tumour that expressed either a control plasmid or V5-UBE2L6-WT. Scale bar: 50 µm.

**Supplementary Figure 5.**


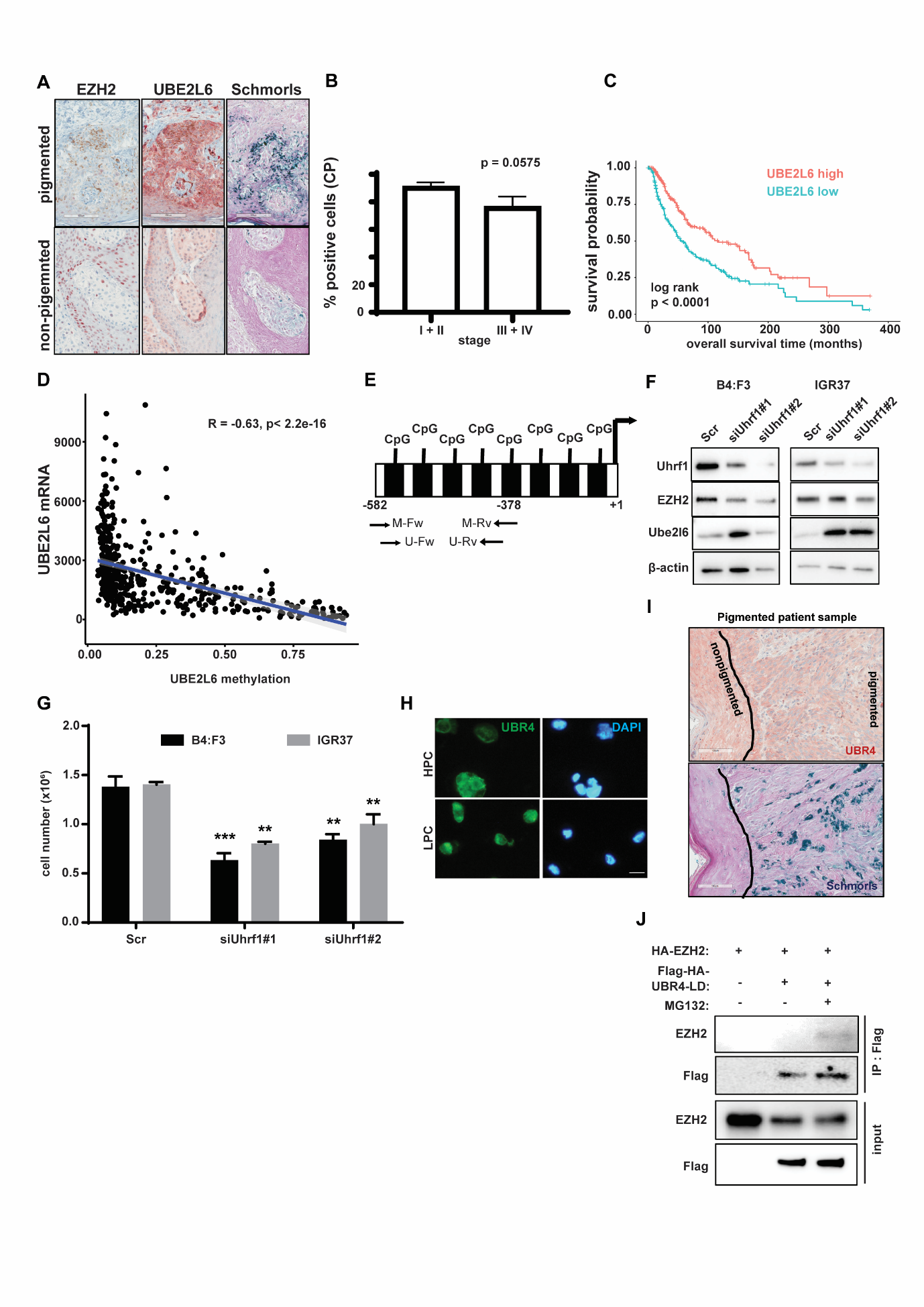


**Supplementary Figure 5. UHRF1 knockdown upregulates UBE2L6 and in turn downregulates EZH2 in melanoma cells.** (A) Immunohistochemical staining of EZH2 and UBE2L6 in representative Schmorl’s-stained pigmented and non-pigmented human melanomas. Scale bar, 50 μm. (B) UBE2L6 expression according to disease staging in 39 patients from the Melanoma Research Victoria cohort. Protein score = the percentage of immune-positive cells. Data from stage I-II (n=22) and stage III-IV (n=17) patients were compared by unpaired student t-test. (C) Kaplan-Meier survival curves of TCGA cutaneous melanoma patients (n = 427 patients), stratified by Ube2l6 mRNA levels. (D) Correlation between *UBE2L6* mRNA and *UBE2L6* methylation in TCGA cutaneous melanomas. (E) Schematic representation of *UBE2L6* promoter at CpG sites spanning from -582 to -378 relative to the transcriptional start site (TSS). Forward primer (Fw) and reverse primers (Rv) used in methylation (m) and unmethylation (um) analysis are depicted. (F) UHRF1, EZH2 and UBE2L6 protein levels evaluated by western blot, and (G) viability by Trypan blue cell counting in 28:B4:F3 and IGR37 cells transfected with scramble or two siUhrf1 oligos. Data derived from three independent experiments are presented as mean ±SD, analyzed by one-way ANOVA plus Tukey’s multiple comparison test. ** p< 0.01, *** p< 0.001. (H) UBR4 IF staining of HPCs and LPCs purified from B16-F10 cells. Scale bar, 10 μm. (I) Immunohistochemical staining of UBR4 in representative Schmorl’s stained pigmented human melanomas. Scale bar, 100 μm (J) HA-tagged EZH2 and Flag-HA-tagged UBR-LD were coexpressed into HEK293 cells maintained ±MG132. Interactions between EZH2 and UBR4-LD were determined by immunoprecipitation with anti-Flag antibody followed by western blotting with anti-EZH2 antibody.
